# Association of endothelial dysfunction and peripheral arterial disease with sarcopenia in chronic kidney disease

**DOI:** 10.1002/jcsm.13471

**Published:** 2024-04-21

**Authors:** Bang‐Gee Hsu, Chih‐Hsien Wang, Yu‐Hsien Lai, Chiu‐Huang Kuo, Yu‐Li Lin

**Affiliations:** ^1^ Division of Nephrology Hualien Tzu Chi Hospital, Buddhist Tzu Chi Medical Foundation Hualien Taiwan; ^2^ School of Medicine Tzu Chi University Hualien Taiwan; ^3^ School of Post‐baccalaureate Chinese Medicine Tzu Chi University Hualien Taiwan

**Keywords:** chronic kidney disease, endothelial biomarkers, endothelial function, peripheral arterial disease, sarcopenia

## Abstract

**Background:**

Endothelial dysfunction and peripheral arterial disease (PAD), which disturb skeletal muscle microperfusion, are highly prevalent in patients with chronic kidney disease (CKD). We evaluated the association of endothelial dysfunction and PAD with sarcopenia in patients with non‐dialysis CKD.

**Methods:**

This cross‐sectional study included 420 patients with stages 3–5 non‐dialysis CKD aged 69.0 ± 11.8 years. Skeletal muscle index (skeletal muscle mass/height^2^), handgrip strength, 6‐m gait speed and strength of hip flexion and knee extension were measured. Sarcopenia was defined according to the Asian Working Group for Sarcopenia 2019. Endothelial dysfunction and PAD were assessed using the vascular reactivity index (VRI) and ankle–brachial index (ABI), respectively. A VRI < 1.0 was classified as poor endothelial function, and an ABI < 0.9 was defined as PAD. Additionally, endothelial and inflammatory biomarkers, including intercellular adhesion molecule‐1 (ICAM‐1), vascular cell adhesion molecule‐1 (VCAM‐1), asymmetric dimethylarginine, endothelin‐1 (ET‐1) and interleukin‐6, were measured in a subgroup of 262 patients.

**Results:**

Among the participants, 103 (24.5%) were classified as having sarcopenia. Compared with patients without sarcopenia, those with sarcopenia had significantly lower ABI (1.04 ± 0.16 vs. 1.08 ± 0.15, *P* = 0.028 for the right ABI; 1.01 ± 0.16 vs. 1.06 ± 0.16, *P* = 0.002 for the left ABI) and VRI (0.83 ± 0.57 vs. 1.08 ± 0.56, *P* < 0.001) and had higher serum levels of ICAM‐1 (*P* < 0.001), VCAM‐1 (*P* = 0.003) and ET‐1 (*P* = 0.037). Multivariate logistic regression revealed that, beyond age and body mass index, the average ABI (odds ratio [OR]: 0.81/0.1 increase; 95% confidence interval [CI]: 0.67–0.98; *P* = 0.032) and VRI (OR: 0.93/0.1 increase; 95% CI: 0.88–0.98; *P* = 0.010) were independently associated with sarcopenia. Among the endothelial biomarkers measured, ICAM‐1 (OR: 2.47/1‐SD increase; 95% CI: 1.62–3.75) and VCAM‐1 (OR: 1.91/1‐SD increase; 95% CI: 1.27–2.87) were independent predictors of sarcopenia. Group stratification based on the cut‐offs of VRI and ABI showed that those with both poor VRI and ABI had the greatest risk for sarcopenia (OR: 4.22; 95% CI: 1.69–10.49), compared with those with normal VRI and ABI.

**Conclusions:**

Endothelial dysfunction and PAD are independently associated with sarcopenia in patients with stages 3–5 CKD, suggesting the dominant role of vascular dysfunction in sarcopenia.

## Introduction

Sarcopenia, defined as an accelerated and progressive loss of both skeletal muscle mass (SMM) and strength, is highly prevalent in patients with chronic kidney disease (CKD), resulting in an increased risk of adverse outcomes that include poor quality of life, falls, hospitalization and mortality.^1^, ^2^, ^3^, ^4^ As CKD progresses, the risk of sarcopenia markedly increases. Beyond the common pathogenesis in geriatric sarcopenia, patients with CKD are susceptible to more CKD‐specific risks of sarcopenia, such as metabolic acidosis, accumulated uremic toxins, overexpression of angiotensin II and protein‐energy malnutrition^5^; additional potential mechanisms remain under investigation.

The burden of vascular dysfunction and the risk of adverse vascular events are considerably high in patients with CKD. Atherosclerosis and endothelial dysfunction are encountered in the early stages of CKD, which further progress as renal function declines.^6^, ^7^ Given the high degree of vascularization of skeletal muscle, disturbances in skeletal muscle microperfusion resulting from atherosclerosis and endothelial dysfunction may be involved in the pathogenesis of sarcopenia in patients with CKD. Previous experimental studies have established the critical role of endothelium‐regulatory vasodilatation on amino acid delivery into skeletal muscle and protein anabolism, whereas blunted vasodilatation results in anabolic resistance.^8^, ^9^, ^10^ Moreover, atherosclerosis of the lower extremities, a characteristic of peripheral arterial disease (PAD), induces skeletal muscle ischaemia and reperfusion injury, resulting in impaired mitochondrial function and enhanced inflammation and oxidative stress.^11^ Unfortunately, despite the frequency of endothelial dysfunction, atherosclerosis and sarcopenia in patients with CKD, their interrelationships remain largely unexplored.

Herein, we primarily evaluated the association of endothelial dysfunction and PAD with sarcopenia in patients with stages 3–5 non‐dialysis CKD. Additionally, the potential link between endothelial and inflammatory biomarkers, including intercellular adhesion molecule‐1 (ICAM‐1), vascular cell adhesion molecule‐1 (VCAM‐1), asymmetric dimethylarginine (ADMA), endothelin‐1 (ET‐1) and interleukin‐6 (IL‐6), and sarcopenia was also evaluated in a subgroup of our CKD cohort.

## Methods

### Study design and participants

This is a cross‐sectional study that analysed the Tzu‐Chi CKD cohort, which is an ongoing prospective study conducted in outpatient CKD clinics at Hualien Tzu‐Chi Medical Center since April 2018 with the aim of evaluating risk factors and potential mechanisms of vascular dysfunction and skeletal muscle wasting in non‐dialysis CKD. In this study, patients aged >20 years and who were diagnosed as having stages 3–5 CKD, which was defined as an estimated glomerular filtration rate (eGFR) < 60 mL/min for at least 3 months, were invited to take part in the study. Patients who had any infection or acute myocardial infarction within 3 months of the study onset, history of stroke, pacemaker implantation, amputated limbs, obvious uremic symptoms or signs, wheelchair‐bound or bed‐ridden, or those who refused to participate were excluded. The basic characteristics and comorbidities, including diabetes mellitus (DM), hypertension (HTN), hyperlipidaemia and smoking status, were collected through electronic records. The study was approved by the Institutional Review Board of Hualien Tzu Chi Hospital (IRB 106‐109‐A). All participants provided signed, informed consent.

### Vascular function assessment

Systolic and diastolic blood pressures (BPs) were measured after resting for 10 min. Pulse pressure was calculated as systolic BP minus diastolic BP. Digital thermal monitoring (Endothelix Inc., Houston, TX, USA) was employed to assess endothelial function, and measurements were performed after an overnight fast of 10 h. We placed the BP cuffs on the bilateral upper arms and fixed the skin temperature sensors on the bilateral index fingers. Then, digital thermal monitoring was performed, which included 3 min of stabilization phase, 2 min of cuff‐inflated phase (to 50 mmHg greater than systolic BP) and 5 min of deflation phase. While the cuff was released, blood flow rushed into the hand, and a temperature rebound in the fingertip could be detected. The VENDYS software was used to calculate the vascular reactivity index (VRI), which assessed the maximum difference between the observed temperature rebound curve and the zero‐reactivity curve.^12^, ^13^ A VRI < 1.0 indicated poor endothelial function.^12^ The ankle–brachial index (ABI) was obtained using an automatic machine (VaSera VS‐1000, Fukuda Denshi Co., Ltd., Tokyo, Japan) to measure both arm and ankle BPs simultaneously with the oscillometric method. The average ABI was calculated as the mean ABI values of both legs. PAD was diagnosed when the ABI was <0.9 in either leg.^14^

In a subpopulation of this cohort, serum endothelial and inflammatory biomarkers, including ICAM‐1 and VCAM‐1 (Quantikine human sICAM‐1 and sVCAM‐1, R&D Systems, Europe Ltd, Abington, UK), ADMA (Immundiagnostik AG, Stubenwald‐Allee 8a, 64625 Bensheim, Germany), ET‐1 (Biomedica immunoassays, Biomedica Medizinprodukte GmbH, Austria) and IL‐6 (Abcam, 152 Grove Street Waltham, MA 02453, USA), were measured using enzyme‐linked immunosorbent assay.

### Anthropometric and skeletal muscle mass measurements

Height and weight were measured, and body mass index (BMI) was calculated. Anthropometric measurements include midarm circumference (MAC), triceps skinfold thickness (TSF), waist circumference (WC) and calf circumference (CC), which were all measured by a flexible, inextensible tape and a skinfold calliper (QuickMedical, Issaquah, WA, USA). Midarm muscular circumference (MAMC) was calculated as MAC (cm) − 3.14 × TSF (cm). We measured the WC at the midpoint between the lower margin of the last rib and the top of the iliac crest, while the CC was measured at the level of the greatest circumference on the lower leg.

A tetrapolar bioelectrical impedance device (Biodynamics® BIA 450 Bioimpedance Analyser, Seattle, WA, USA) with a delivery frequency of 50 kHz at 800 mA was used to measure body composition with the patients in the supine position. We estimated SMM based on a well‐validated equation developed by Janssen et al.,^15^ which showed good correlation and agreement with magnetic resonance imaging‐measured SMM in the Taiwanese population^16^:

$$\mathrm{SMM}=\left(\;\frac{{\text{height}}^{2}}{\text{resistance}}\times 0.401\;\right)+\mathrm{age}\times \left(-0.071\right)+\mathrm{sex}\times 3.825+5.102.$$

In the equation, height is in cm; resistance is in ohms; age is in years; and sex: female = 0, male = 1.

SMM (kg) and fat tissue mass (kg) were divided by height squared (m^2^) to generate the skeletal muscle index (SMI) and fat tissue index (FTI). Low SMI was defined as an SMI < 8.87 kg/m^2^ in men and 6.42 kg/m^2^ in women based on 2 standard deviations below the gender‐specific mean of young Taiwanese adults.^16^

Extracellular water (ECW), intracellular water and total body water (TBW) were also obtained from the bioelectrical impedance device. The ECW/TBW ratio was calculated to indicate hydration status.^17^

### Skeletal muscle strength and usual gait speed measurements

Maximal handgrip strength (HGS) was measured bilaterally using a handheld dynamometer (Jamar Plus Digital Hand Dynamometer, SI Instruments Pty Ltd, Hilton, Australia). Patients were instructed to hold the dynamometer with maximal force in the standing position with the arm fixed at right angles and the elbow at the side of the body. Three measurements were performed, with a 1‐min resting interval between each measurement. Low HGS was defined as an average HGS of <28 kg in men and 18 kg in women based on the Asian Working Group for Sarcopenia (AWGS) 2019 consensus.^18^

With the patients in a sitting position and the knee flexed at a right angle, a portable force evaluation and testing dynamometer (MicroFET2®, Hogan Health Industries, Inc., UT, USA)^19^, ^20^ was used to assess the muscle strength of the lower extremities. First, the device was placed between the operator's hand and the patient's limb to be tested. Then, patients were instructed to apply force as hard as possible against the dynamometer pad during the 5‐s test period by raising their legs and straightening their knees. The procedure was repeated three times, with a 1‐min resting interval between each measurement. The average value of both extremities was calculated for further analysis.

For gait speed (GS), patients were instructed to walk on a flat and straight 6‐m path at their usual speed. GS was then calculated as distance (6 m) divided by time (seconds). The test was not performed on 23 (5.5%) patients who reported dizziness, pain or any difficulty with usual walking. Slow GS was defined as a GS of <1.0 m/s based on the AWGS 2019 consensus.^18^

### Sarcopenia diagnosis

Patients who fulfilled the criteria of low SMI with either low HGS or slow GS were diagnosed as having sarcopenia based on the AWGS 2019 consensus.^18^

### Biochemistry data

Routine biochemistry data, including serum creatinine, albumin (bromocresol green method) and urine protein‐to‐creatinine ratio (UPCR), were measured in all patients using an autoanalyser (Siemens Advia 1800, Siemens Healthcare GmbH, Henkestr, Germany). eGFR was calculated according to the Modification of Diet in Renal Disease formula.^21^

### Statistical analysis

The normality of continuous variables was assessed using the Kolmogorov–Smirnov test. Variables with a normal distribution were expressed as mean ± standard deviation, whereas those not normally distributed were expressed as medians and interquartile ranges. Differences between groups were tested using the Student's independent *t* test or the Mann–Whitney *U* test. Categorical variables were expressed as absolute numbers and relative frequency (%) and analysed by the *χ* ^2^ test. Univariate and multivariate logistic regression analyses were conducted to evaluate the independent association of endothelial function, its related serum biomarkers and PAD with sarcopenia. Data were analysed using SPSS for Windows (Version 19.0; SPSS Inc., Chicago, IL, USA). *P* values < 0.05 were considered statistically significant.

## Results

Overall, 420 patients (258 men and 162 women) with CKD, with a mean age of 69.0 ± 11.8 years, were enrolled in the study. The study flow diagram is shown in *Figure* *S1*. Of the patients, 42.9%, 40.2% and 16.9% had CKD stages 3, 4 and 5, respectively. The proportion of patients with DM, HTN and hyperlipidaemia was 61.4%, 74.8% and 35.5%, respectively. Among the participants, 103 (24.5%) were classified as having sarcopenia. The mean SMI value was 8.7 ± 1.7 kg/m^2^. The characteristics of all participants are shown in *Table* *1*. Patients with sarcopenia were older (*P* < 0.001), had a lower prevalence of DM (*P* = 0.017) and had significantly lower values for anthropometric data (weight, BMI, SMI, MAC, MAMC, WC and CC, all *P* < 0.001), UPCR (*P* = 0.012) and diastolic BP (*P* = 0.046) while having a higher pulse pressure (*P* = 0.043). Notably, those with sarcopenia had significantly lower ABI (1.04 ± 0.16 vs. 1.08 ± 0.15, *P* = 0.028 for the right ABI; 1.01 ± 0.16 vs. 1.06 ± 0.16, *P* = 0.002 for the left ABI) and VRI (0.83 ± 0.57 vs. 1.08 ± 0.56, *P* < 0.001) and higher serum levels of ICAM‐1 (*P* < 0.001), VCAM‐1 (*P* = 0.003) and ET‐1 (*P* = 0.037). The associations between smoking and the ECW/TBW ratio on vascular function and relevant variables were also reported in *Tables* *S1* and *S2*.

**Table 1:** Demographic and clinical characteristics of 420 chronic kidney disease patients

Characteristics	All (*n* = 420)	Non‐sarcopenia (*n* = 317)	Sarcopenia (*n* = 103)	*P*
Demographics				
Age (years)	69.0 ± 11.8	66.6 ± 11.7	76.2 ± 9.0	<0.001*
Female, *n* (%)	162 (38.6)	130 (41.0)	32 (31.1)	0.072
Current smoker, *n* (%)	65 (15.5)	54 (17.0)	11 (10.7)	0.121
DM, *n* (%)	258 (61.4)	205 (64.7)	53 (51.5)	0.017*
HTN, *n* (%)	314 (74.8)	23 (73.5)	81 (78.6)	0.297
Hyperlipidaemia, *n* (%)	149 (35.5)	117 (36.9)	32 (31.1)	0.282
CKD stages				
Stage 3	180 (42.9)	128 (40.4)	52 (50.5)	0.080
Stage 4	169 (40.2)	129 (40.7)	40 (38.8)	
Stage 5	71 (16.9)	60 (18.9)	11 (10.7)	
Anthropometric data				
Height (cm)	160.7 ± 8.3	160.8 ± 8.4	160.2 ± 7.8	0.558
Weight (kg)	67.0 ± 12.9	69.6 ± 12.9	59.1 ± 9.6	<0.001*
BMI (kg/m^2^)	25.9 ± 4.2	26.8 ± 4.0	23.0 ± 3.1	<0.001*
SMI (kg/m^2^)	8.7 ± 1.7	9.1 ± 1.7	7.4 ± 1.1	<0.001*
MAC (cm)	27.7 ± 3.3	28.4 ± 3.2	25.7 ± 2.6	<0.001*
MAMC (cm)	22.5 ± 3.0	22.9 ± 3.1	21.1 ± 2.4	<0.001*
WC (cm)	90.5 ± 11.6	92.1 ± 11.7	85.3 ± 9.5	<0.001*
CC (cm)	34.1 ± 3.7	35.1 ± 3.5	31.2 ± 2.0	<0.001*
ECW/TBW (%)	48 (45–51)	48 (45–51)	48 (46–51)	0.538
Laboratory data				
Creatinine (mg/dL)	2.3 (1.7–3.2)	2.3 (1.7–3.3)	2.2 (1.6–3.0)	0.166
eGFR (mL/min)	27.6 (17.8–37.8)	27.1 (17.2–37.8)	30.2 (20.3–38.0)	0.116
Haemoglobin (g/dL)	11.5 ± 2.1	11.6 ± 2.2	11.3 ± 2.0	0.218
Albumin (g/dL)	4.1 (3.8–4.3)	4.1 (3.8–4.3)	4.1 (3.9–4.2)	0.612
UPCR (g/g)	0.5 (0.2–1.7)	0.6 (0.2–1.8)	0.3 (0.1–0.9)	0.012*
Vascular function				
Systolic BP (mmHg)	132 ± 18	132 ± 18	133 ± 20	0.665
Diastolic BP (mmHg)	80 ± 12	81 ± 11	78 ± 12	0.046*
Pulse pressure (mmHg)	52 ± 15	51 ± 15	55 ± 15	0.043*
Right ABI	1.07 ± 0.15	1.08 ± 0.15	1.04 ± 0.16	0.028*
Left ABI	1.05 ± 0.16	1.06 ± 0.16	1.01 ± 0.16	0.002*
VRI	1.01 ± 0.57	1.08 ± 0.56	0.83 ± 0.57	<0.001*
Biomarkers^a^				
ICAM‐1 (ng/mL)	226 (175–269)	215 (167–257)	255 (201–286)	<0.001*
VCAM‐1 (ng/mL)	3066 (2751–3436)	2998 (2657–3357)	3174 (2945–3549)	0.003*
ADMA (μmol/L)	0.58 (0.50–0.70)	0.58 (0.49–0.70)	0.61 (0.53–0.70)	0.118
ET‐1 (pmol/L)	0.42 (0.27–0.57)	0.40 (0.26–0.55)	0.49 (0.29–0.63)	0.037*
IL‐6 (pg/mL)	5.78 (3.69–9.49)	5.61 (3.65–8.88)	6.87 (4.19–11.07)	0.072

Abbreviations: ABI, ankle–brachial index; ADMA, asymmetric dimethylarginine; BMI, body mass index; BP, blood pressure; CC, calf circumference; CKD, chronic kidney disease; DM, diabetes mellitus; ECW/TBW, extracellular water/total body water; eGFR, estimated glomerular filtration rate; ET‐1, endothelin‐1; HTN, hypertension; ICAM‐1, intercellular adhesion molecule‐1; IL‐6, interleukin‐6; MAC, midarm circumference; MAMC, midarm muscular circumference; SMI, skeletal muscle index; UPCR, urine protein‐to‐creatinine ratio; VCAM‐1, vascular cell adhesion molecule‐1; VRI, vascular reactivity index; WC, waist circumference.

^a^ *n* = 262.

* *P* < 0.05 was considered significant.

**Figure 1:**
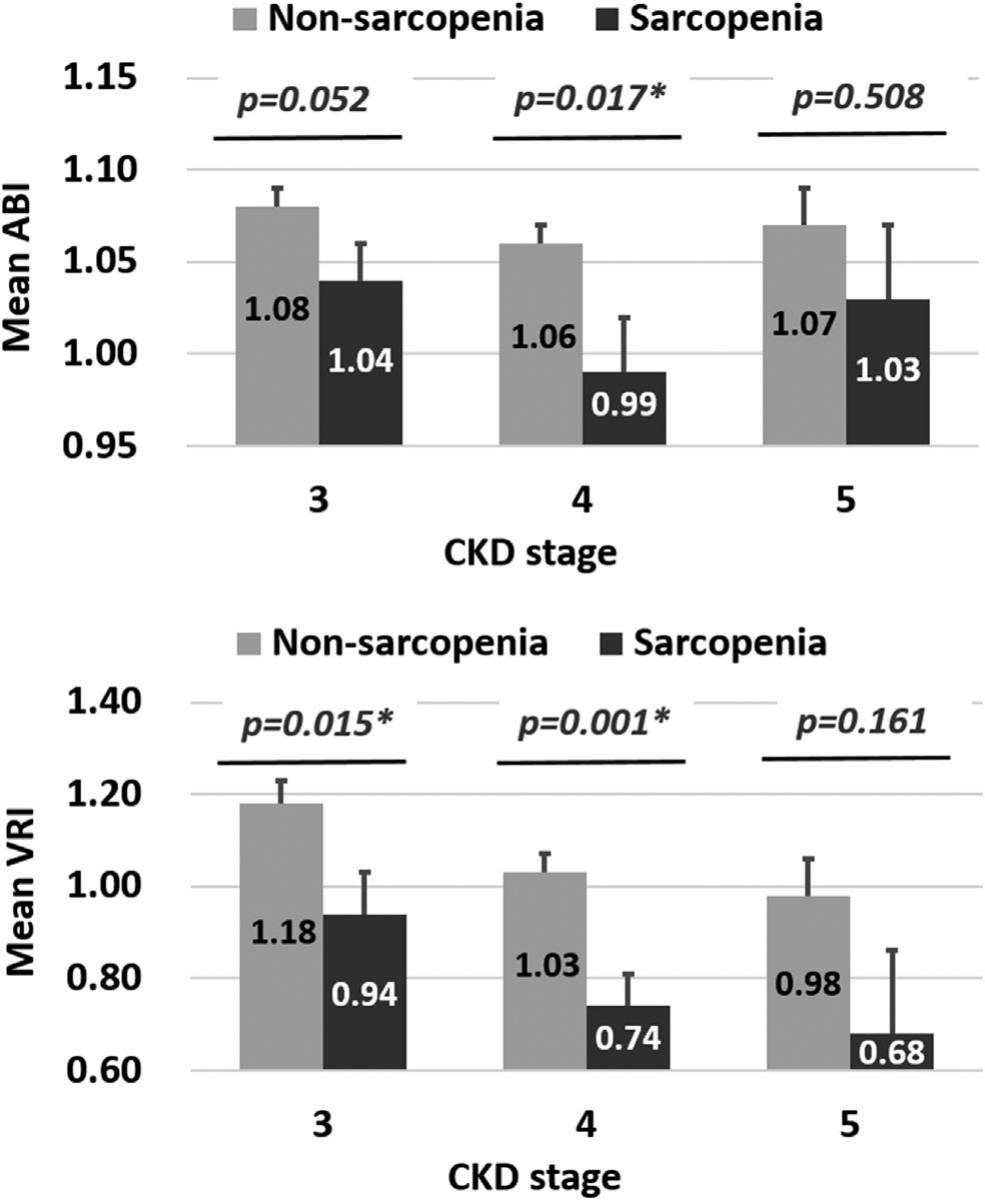
Mean ankle–brachial index (ABI) and vascular reactivity index (VRI) values between patients with and without sarcopenia. Error bars represent standard errors. *P < 0.005. CKD, chronic kidney disease.

The mean values of ABI and VRI between patients with CKD with and without sarcopenia are depicted in *Figure* *1*. Among patients with CKD stages 3 and 4, those with sarcopenia tended to have lower average ABI and VRI values compared with those without sarcopenia. However, these differences were not significant among patients with CKD stage 5.

The comparison of anthropometric and skeletal muscle metrics between patients with CKD with and without poor endothelial function and PAD is presented in *Table* *2*. Regarding anthropometric measurements, there was no significant difference between patients with and without poor endothelial function and PAD; however, CC was lower in those with poor endothelial function (*P* = 0.001) and those with PAD (*P* = 0.004). Regarding skeletal muscle measurements, patients with poor endothelial function had significantly lower SMI (*P* = 0.024), HGS (*P* = 0.046) and GS (*P* < 0.001) and marginally weaker hip flexion (*P* = 0.061). Meanwhile, those with PAD had significantly lower SMI (*P* = 0.006), HGS (*P* = 0.008) and GS (*P* < 0.001) and weaker hip flexion (*P* = 0.001) and knee extension (*P* < 0.001).

**Table 2:** Comparisons of anthropometric and skeletal muscle metrics between patients with chronic kidney disease with and without poor endothelial function and peripheral arterial disease (*n* = 420)

Characteristics	Poor endothelial function		*P*	PAD		*P*
	Absence (*n* = 224)	Presence (*n* = 196)		Absence (*n* = 355)	Presence (*n* = 65)	
Anthropometric data						
Height (cm)	160.9 ± 7.9	160.4 ± 8.7	0.604	160.9 ± 8.3	159.2 ± 8.4	0.115
Weight (kg)	67.7 ± 12.8	66.3 ± 13.1	0.252	67.2 ± 13.3	66.1 ± 11.1	0.538
BMI (kg/m^2^)	26.1 ± 4.1	25.7 ± 4.2	0.283	25.9 ± 4.2	26.1 ± 3.6	0.731
MAC (cm)	27.9 ± 3.4	27.5 ± 3.1	0.131	27.7 ± 3.3	27.7 ± 3.3	0.959
MAMC (cm)	22.3 ± 3.3	22.6 ± 2.7	0.434	22.5 ± 3.0	22.4 ± 3.0	0.933
WC (cm)	89.6 ± 11.8	91.4 ± 11.2	0.107	90.4 ± 11.8	90.7 ± 10.6	0.857
CC (cm)	34.7 ± 3.6	33.5 ± 3.7	0.001*	34.4 ± 3.7	32.9 ± 3.2	0.004*
Skeletal muscle metrics						
SMI (kg/m^2^)	8.9 ± 1.7	8.5 ± 1.8	0.024*	8.8 ± 1.7	8.2 ± 1.6	0.006*
FTI (kg/m^2^)	8.0 ± 2.7	8.1 ± 2.6	0.665	7.9 ± 2.7	8.5 ± 2.5	0.122
HGS (kg)	24.6 ± 8.0	23.0 ± 8.6	0.046*	24.3 ± 8.2	21.4 ± 8.3	0.008*
GS (m/s)^a^	0.90 ± 0.27	0.80 ± 0.27	<0.001*	0.88 ± 0.27	0.72 ± 0.24	<0.001*
Hip flexion strength (kg‐force)	18.3 ± 6.8	17.1 ± 6.3	0.061	18.2 ± 6.6	15.2 ± 6.1	0.001*
Knee extension strength (kg‐force)	15.6 ± 5.8	15.1 ± 5.4	0.402	15.8 ± 5.7	13.3 ± 4.4	<0.001*

Abbreviations: BMI, body mass index; CC, calf circumference; FTI, fat tissue index; GS, gait speed; HGS, handgrip strength; MAC, midarm circumference; MAMC, midarm muscular circumference; PAD, peripheral arterial disease; SMI, skeletal muscle index; WC, waist circumference.

^a^ Twenty‐three patients (5.5%) did not perform gait speed tests.

* *P* < 0.05 was considered significant.

*Table* *3* demonstrates the results of univariate and multivariate logistic regression of sarcopenia among patients with CKD. After adjusting for confounding factors, including age, sex, DM, BMI, eGFR, albumin, UPCR, pulse pressure, ABI and VRI, multivariate analysis revealed that age (odds ratio [OR]: 1.12; 95% confidence interval [CI]: 1.08–1.16; *P* < 0.001), BMI (OR: 0.68; 95% CI: 0.61–0.77; *P* < 0.001), average ABI (OR: 0.81/0.1 increase; 95% CI: 0.67–0.98; *P* = 0.032) and VRI (OR: 0.93/0.1 increase; 95% CI: 0.88–0.98; *P* = 0.010) were independently associated with sarcopenia. Further adjusting the ECW/TBW ratio did not alter the association between vascular dysfunction and sarcopenia (*Table* *S3*). The subgroup analyses were also provided in *Figures* *S2* and *S3*.

**Table 3:** Univariate and multivariate logistic regression analysis of factors associated with sarcopenia in patients with chronic kidney disease (*n* = 420)

Variable	Univariate		Multivariate	
	OR (95% CI)	*P* value	OR (95% CI)	*P* value
Age (years)	1.10 (1.07–1.13)	<0.001*	1.12 (1.08–1.16)	<0.001*
Female	0.65 (0.40–1.04)	0.073	0.83 (0.44–1.57)	0.573
DM	0.58 (0.37–0.91)	0.017*	0.97 (0.48–1.68)	0.902
BMI (kg/m^2^)	0.73 (0.67–0.79)	<0.001*	0.68 (0.61–0.77)	<0.001*
eGFR (mL/min)	1.01 (0.99–1.03)	0.132	1.02 (0.99–1.05)	0.112
Albumin (g/dL)	1.04 (0.59–1.83)	0.902	1.21 (0.47–3.13)	0.699
UPCR (g/g)	0.95 (0.86–1.04)	0.285	1.08 (0.94–1.25)	0.271
Pulse pressure (mmHg)	1.02 (1.00–1.03)	0.044*	0.99 (0.97–1.01)	0.277
Average ABI (per 0.1 increase)	0.81 (0.70–0.93)	0.004*	0.81 (0.67–0.98)	0.032*
VRI (per 0.1 increase)	0.92 (0.89–0.96)	<0.001*	0.93 (0.88–0.98)	0.010*

*Note*: In the multivariate models, all variables were adjusted together. Abbreviations: ABI, ankle–brachial index; BMI, body mass index; CI, confidence interval; DM, diabetes mellitus; eGFR, estimated glomerular filtration rate; OR, odds ratio; UPCR, urine protein‐to‐creatinine ratio; VRI, vascular reactivity index.

* *P* < 0.05 was considered significant.

Regarding the different clinical relevance of muscle mass, strength and physical performance, the independent associations between VRI, average ABI and individual sarcopenia components were further explored, with the results showing that the average ABI was independently associated with a low SMI (OR: 0.78/0.1 increase; 95% CI: 0.65–0.94; *P* = 0.009) and that VRI was independently associated with a low SMI (OR: 0.91/0.1 increase; 95% CI: 0.86–0.95; *P* < 0.001) and slow GS (OR: 0.95/0.1 increase; 95% CI: 0.91–0.99; *P* = 0.018) (*Table* *4*).

**Table 4:** Univariate and multivariate logistic regression analysis of the association of vascular reactivity index and average ankle–brachial index with low skeletal muscle index, handgrip strength and slow gait speed (*n* = 420)

Dependent variable	Univariate		Multivariate	
	OR (95% CI)	*P* value	OR (95% CI)	*P* value
Average ABI (per 0.1 increase)				
Low SMI	0.83 (0.72–0.95)	0.007*	0.78 (0.65–0.94)	0.009*
Low HGS	0.91 (0.80–1.04)	0.146	0.97 (0.84–1.12)	0.672
Slow GS^a^	0.80 (0.68–0.95)	0.009*	0.89 (0.75–1.07)	0.218
VRI (per 0.1 increase)				
Low SMI	0.92 (0.88–0.96)	<0.001*	0.91 (0.86–0.95)	<0.001*
Low HGS	0.96 (0.93–0.99)	0.014*	0.98 (0.95–1.02)	0.372
Slow GS^a^	0.93 (0.89–0.97)	<0.001*	0.95 (0.91–0.99)	0.018*

*Note*: In the multivariate models, age, gender, diabetes mellitus, body mass index, estimated glomerular filtration rate, albumin, urine protein‐to‐creatinine ratio and pulse pressure were adjusted. Abbreviations: ABI, ankle–brachial index; CI, confidence interval; GS, gait speed; HGS, handgrip strength; OR, odds ratio; SMI, skeletal muscle index; VRI, vascular reactivity index.

^a^ Twenty‐three patients (5.5%) did not perform gait speed tests.

* *P* < 0.05 was considered significant.

**Figure 2:**
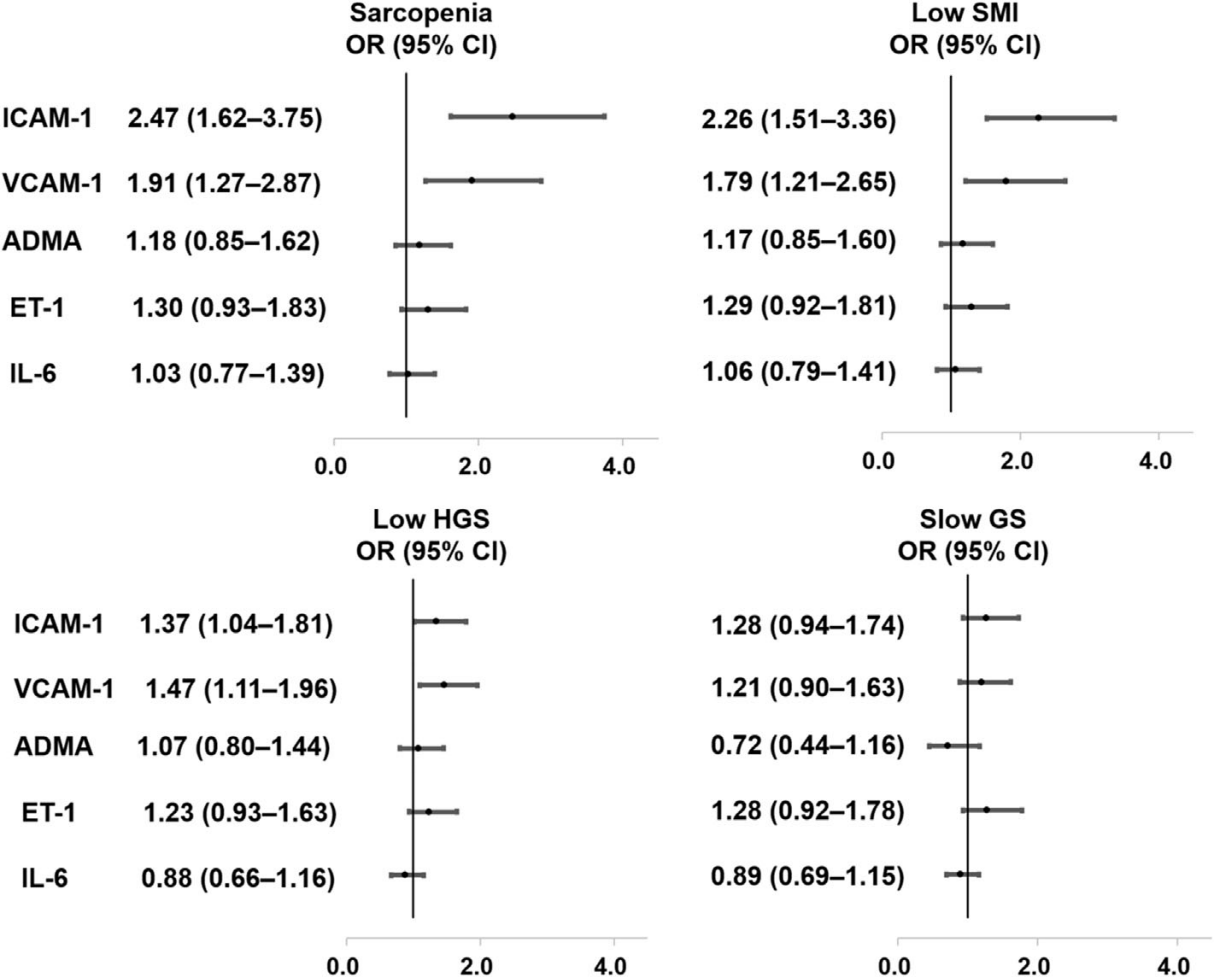
Associations of endothelial biomarkers (per 1‐SD increase) with sarcopenia, low skeletal muscle index (SMI), handgrip strength (HGS) and slow gait speed (GS) in a subgroup of 262 patients with chronic kidney disease after adjusting for age, gender, diabetes mellitus, body mass index, estimated glomerular filtration rate, albumin, urine protein‐to‐creatinine ratio, pulse pressure and interleukin‐6 (IL‐6). ADMA, asymmetric dimethylarginine; CI, confidence interval; ET‐1, endothelin‐1; ICAM‐1, intercellular adhesion molecule‐1; OR, odds ratio; VCAM‐1, vascular cell adhesion molecule‐1.

Associations of endothelial and inflammatory biomarkers with sarcopenia and its individual components, which were analysed in our subgroup of 262 participants, are depicted in *Figure* *2*. After full adjustment, ICAM‐1 and VCAM‐1 were associated with sarcopenia, low SMI and HGS.

**Figure 3:**
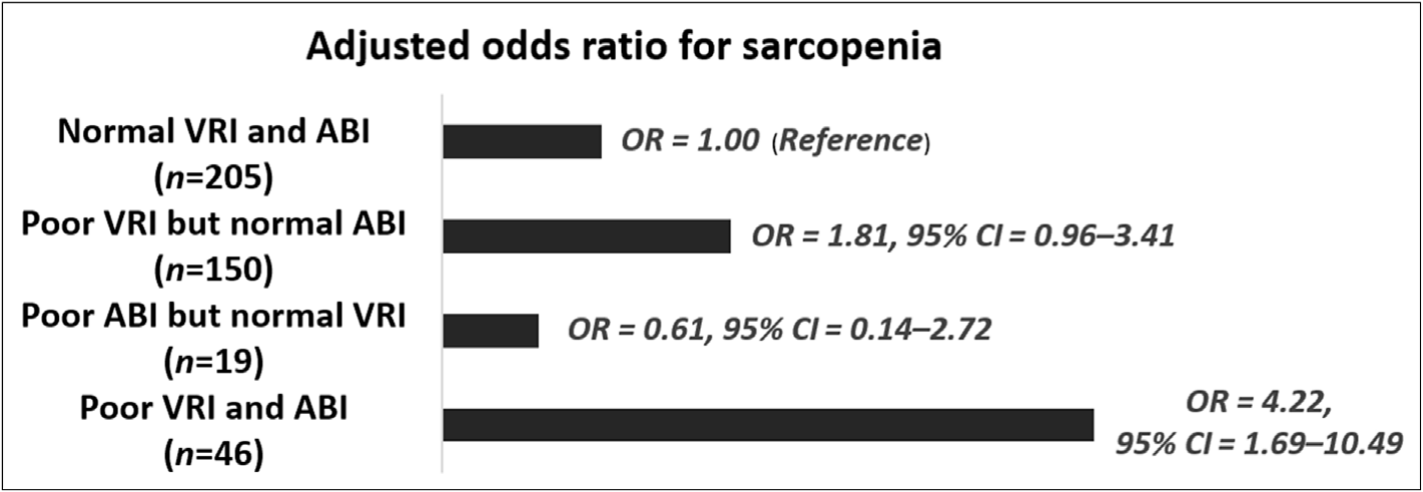
Combined effects of poor vascular reactivity index (VRI) and ankle–brachial index (ABI) on the risk of sarcopenia (*n* = 420). The model was fully adjusted for age, gender, diabetes mellitus, body mass index, estimated glomerular filtration rate, albumin, urine protein‐to‐creatinine ratio and pulse pressure. CI, confidence interval; OR, odds ratio.

*Figure* *3* depicts the combined effects of poor VRI and ABI on the risk of developing sarcopenia. Compared with participants with normal VRI and ABI, those with poor VRI and ABI had the greatest risk for developing sarcopenia (OR: 4.22; 95% CI: 1.69–10.49).

## Discussion

The main finding of our study was that endothelial dysfunction and PAD, as determined by VRI, endothelial biomarkers and ABI, were associated with sarcopenia in patients with non‐dialysis CKD, indicating the critical role of vascular dysfunction in the development of sarcopenia in CKD.

The maintenance of skeletal muscle health is determined by the balance of synthesis and breakdown of skeletal muscle protein, which partially depends on the integrity of skeletal muscle microcirculation. Endothelial dysfunction reduces muscle protein synthesis by compromising the delivery of nutrients into skeletal muscles and hampering their utility,^8^, ^9^, ^10^, ^22^ whereas ischaemia and reperfusion injury resulting from lower extremity atherosclerosis accelerate muscle protein degradation through mitochondria dysfunction, enhancement of reactive oxygen species and oxidative stress and activation of inflammatory and the transforming growth factor‐beta (TGF‐β) pathway.^11^, ^23^ Observational studies have reported a close relationship between endothelial dysfunction or PAD and sarcopenia in various populations. In elderly adults, flow‐mediated dilation was correlated with SMM,^24^ and elevated serum ADMA levels were associated with weaker handgrip and quadriceps strength and slower GS.^25^ In a large‐scale study from the National Health and Nutrition Examination Survey (1999–2002), older adults with an ABI of <0.9 had lower peak leg force and habitual GS and had an increased odds risk for self‐reported functional dependence.^26^ Another case–control study comparing older adults with and without PAD showed that lower ABI values were associated with lower plantarflexion and knee extension strength but not with HGS.^27^ In community‐dwelling older women, those with endothelial dysfunction, as indicated by a low reactive hyperaemia index, had weaker HGS than those with normal endothelial function.^28^ Our previous study on patients who underwent renal transplantation showed that endothelial dysfunction, as determined by VRI, was significantly associated with sarcopenia and slow GS.^29^

In line with the previous studies, this work demonstrated that in patients with stages 3–5 CKD, both endothelial dysfunction and PAD were independent predictors of sarcopenia. Furthermore, after stratifying the patients with CKD based on the cut‐off values of VRI and ABI, we observed that, compared with participants with both normal VRI and ABI, those with preclinical endothelial dysfunction alone exhibited a 1.8‐fold increase in the OR for sarcopenia, which further increased to 4.2‐fold when PAD was established. This finding may implicate the impact of the burden and severity of vascular diseases on the risk of sarcopenia.

Among the endothelial biomarkers measured, ICAM‐1 and VCAM‐1, two members of the Ig‐like supergene family of adhesion molecules normally expressed by endothelial cells, were not only strongly associated with sarcopenia but also correlated with individual diagnostic components, that is, low SMI and HGS. Although these endothelial biomarkers were upregulated by cytokine stimulation,^30^ their association with sarcopenia remained unchanged after adjustment, which included IL‐6 in the models. This indicates that ICAM‐1 and VCAM‐1 are independent endothelial biomarkers of sarcopenia in CKD.

Notably, among the anthropometric measurements, CC was significantly lower in patients with poor endothelial function and in those with PAD. As CC is one of the most powerful anthropometric indices that predicts SMM,^31^, ^32^ this observation supports our main findings regarding the close association between vascular function and SMM in CKD.

Several potential mechanisms could explain the relationship between vascular dysfunction and sarcopenia. First, insulin resistance, a well‐established cause of muscle wasting, may mediate the relationship between endothelial dysfunction and sarcopenia. Through stimulating nitric oxide production to promote skeletal muscular vasodilatation, insulin signalling facilitates the delivery of amino acids and activates the mammalian target of rapamycin complex 1 (mTORC1) pathway, which is crucial for muscle protein anabolic response.^33^ Second, emerging research has reported the critical role of gut‐derived uremic toxins, such as indoxyl sulfate (IS), p‐cresyl sulfate and trimethylamine N‐oxide, in the pathogenesis of endothelial dysfunction.^34^ Among them, IS accumulation was recently reported as contributing to uremic sarcopenia through mitochondria dysfunction, endoplasmic reticulum disturbance, muscle atrophy‐related gene overexpression and impaired angiogenesis via Wnt/β‐catenin axis suppression, as evidenced in vitro and in animal studies.^35^, ^36^, ^37^ We speculated that there might be an indirect pathway between gut‐derived uremic toxins and sarcopenia through the suppression of endothelium‐mediated skeletal muscle vasodilation. Finally, the link between the vascular and muscle systems may also be bidirectional, which can be partially mediated through myokines that are composed of hundreds of peptides secreted from skeletal muscle in response to muscle contraction and stress.^38^ The potential role of myokines in the regulation of the vascular system has been suggested by some in vitro studies. Follistatin‐related protein 1 regulates endothelial cell function and blood vessel growth in skeletal muscles through a nitric oxide synthase‐dependent mechanism,^39^ whereas irisin repairs high glucose‐induced vascular endothelial cell injury and promotes angiogenesis by activating the Notch pathway.^40^ However, further studies are needed to address these important issues.

As sarcopenia and vascular dysfunction are both prevalent in aging^41^ and the mean age of our participant was 69.0 years, it would be difficult to determine if the associations were due to aging or CKD. However, in our subgroup analysis stratified by age, the trend relationships of VRI and ABI with sarcopenia were maintained among those with an age < 65 years.

Chronic inflammation is well established to play a key role in the pathogenesis of muscle catabolism.^42^ However, in our study, there was a lack of association between serum IL‐6 levels and sarcopenia. Due to the cross‐sectional nature of this study, the chronicity of inflammation cannot be determined. This could unintentionally prejudice the result by revealing important roles for inflammation. In addition, the measurement of inflammatory markers was not as extensive as endothelial measurements, and serum C‐reactive protein and tumour necrosis factor‐α were not included.

This is the first study to demonstrate a significant relationship between endothelial dysfunction, PAD and sarcopenia in patients with non‐dialysis CKD, adopting a variety of methods for vascular and skeletal muscle assessment. The exclusion of patients with a number of underlying illnesses and debilities might actually have reduced the strength of the association between vascular dysfunction and sarcopenia. However, several limitations should be acknowledged. First, the proportion of patients with stage 5 CKD was relatively small, and this could explain the non‐significant differences in the mean VRI and ABI values between patients with and without sarcopenia in this stage. Additionally, the unexpectedly lower prevalence of sarcopenia in patients with stage 5 CKD in this study could be attributed to patient selection; patients with sarcopenia and stage 5 CKD frequently presented with clinical illnesses and frailty, decreasing the likelihood of enrolment. Second, potential confounding factors, such as gut‐derived uremic toxins, insulin sensitivity, acid–base status and the amount and sources of daily protein intake, were not assessed in this study. Third, we used a single‐frequency bioelectrical impedance analysis (BIA), and hydration status may not be reliably assessed. Also, the measurement of SMM may be overestimated by hydration status in patients with CKD. However, a recent validation study showed that single‐frequency BIA had good correlation and agreement with dual‐energy X‐ray absorptiometry in patients on dialysis, which was more accurate than multifrequency BIA.^43^ Fourth, in the stratification analysis, very few patients were classified in the poor ABI but normal VRI group because endothelial dysfunction usually develops in the early phase of atherosclerosis.^44^ Also, there were some overlapped characteristics of vascular indices and endothelial biomarkers we measured (*Table* *S4*). Fifth, other endothelial function tests, such as flow‐mediated dilation and forearm plethysmography, were not measured.^45^ In addition, we did not measure cardiac biomarkers, such as B‐type natriuretic peptides, or assess cardiovascular risk scores. Finally, the causal relationship between vascular function and sarcopenia and the underlying biological mechanisms cannot be established in our study.

In conclusion, our study provides important insights regarding the close relationship of endothelial dysfunction and PAD with sarcopenia in patients with stages 3–5 CKD, whose results were reinforced by measuring serum biomarkers in our subgroup. Beyond the well‐established role of exercise on vascular function modulation, whether some other strategies with potential vasodilation or antiatherogenic effects, such as medications and nutrient supplements, mitigate the progression of sarcopenia in CKD should be investigated in further studies.

## Conflict of interest

None was declared by all authors.

## Supporting information


**Figure S1.** The flow diagram of patient enrollment.
**Figure S2.** The association of VRI (per 0.1 increase) and sarcopenia in subgroup analysis, adjusting for age, gender, DM, BMI, eGFR, albumin, UPCR, pulse pressure, and average ABI.
**Figure S3.** The association of average ABI (per 0.1 increase) and sarcopenia in subgroup analysis, adjusting for age, gender, DM, BMI, eGFR, albumin, UPCR, pulse pressure, and VRI.
**Table S1.** Vascular function and serum biomarkers between current smoker and non‐smoker.
**Table S2.** Correlation of ECW/TBW ratio with HTN, renal function, VRI, ABI, endothelial markers, and interleukin‐6.
**Table S3.** Multivariate logistic regression analysis of factors associated with sarcopenia, further adjusting for ECW/TBW ratio.
**Table S4.** Correlation matrix of vascular function tests, endothelial markers, and interleukin‐6.
