# Supplementary material for: Association of endothelial dysfunction and peripheral arterial disease with sarcopenia in chronic kidney disease
Source: J Cachexia Sarcopenia Muscle. 2024 Apr 21;15(3):1199–208. doi: 10.1002/jcsm.13471 (PMC11154745; doi:10.1002/jcsm.13471)
Supplement: Supplementary file 1 — Figure S1. The flow diagram of patient enrollment. Figure S2. The association of VRI (per 0.1 increase) and sarcopenia in subgroup analysis, adjusting for age, gender, DM, BMI, eGFR, albumin, UPCR, pulse pressure, and average ABI. Figure S3. The association of average ABI (per 0.1 increase) and sarcopenia in subgroup analysis, adjusting for age, gender, DM, BMI, eGFR, albumin, UPCR, pulse pressure, and VRI. Table S1. Vascular function and serum biomarkers between current smoker and non‐smoker. Table S2. Correlation of ECW/TBW ratio with HTN, renal function, VRI, ABI, endothelial markers, and interleukin‐6. Table S3. Multivariate logistic regression analysis of factors associated with sarcopenia, further adjusting for ECW/TBW ratio. Table S4. Correlation matrix of vascular function tests, endothelial markers, and interleukin‐6. [file JCSM-15-1199-s001.docx]

**Figure S1. The flow diagram of patient enrollment.**


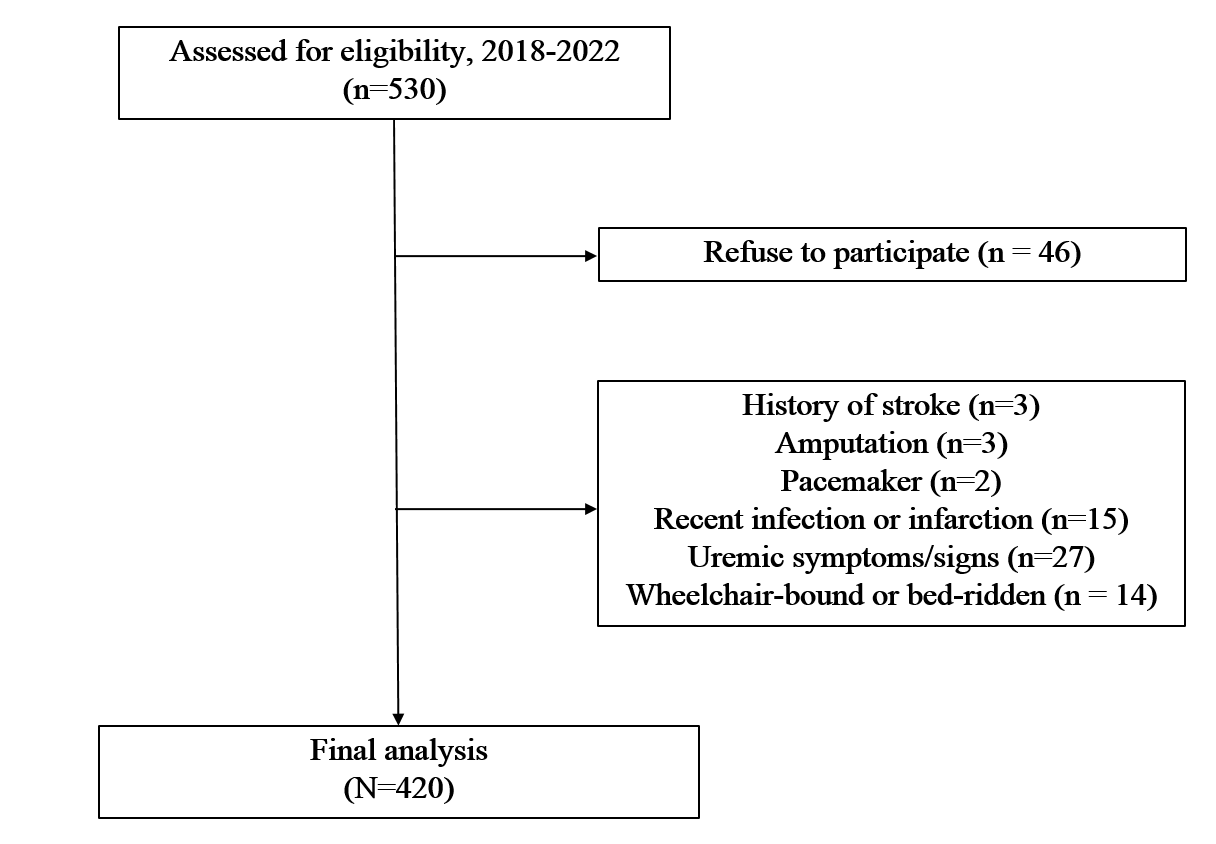


**Figure S2. The association of VRI (per 0.1 increase) and sarcopenia in subgroup analysis, adjusting for age, gender, DM, BMI, eGFR, albumin, UPCR, pulse pressure, and average ABI.**


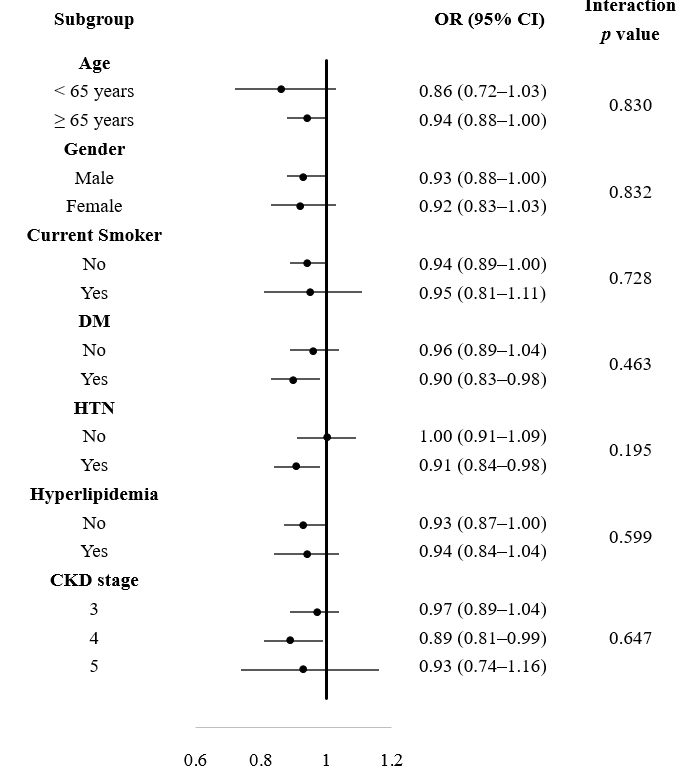


**Figure S3. The association of average ABI (per 0.1 increase) and sarcopenia in subgroup analysis, adjusting for age, gender, DM, BMI, eGFR, albumin, UPCR, pulse pressure, and VRI.**


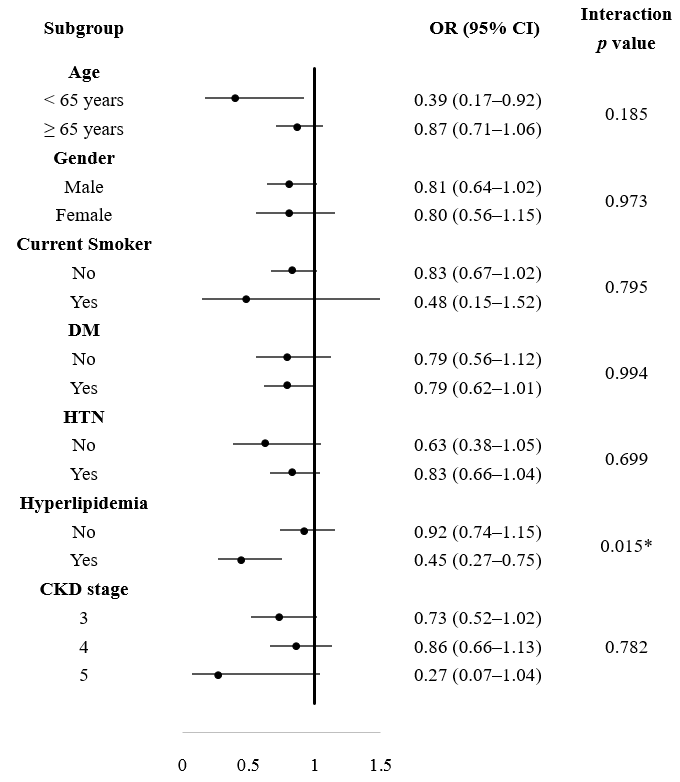


**Table S1. Vascular function and serum biomarkers between current smoker and non-smoker.**

| **Characteristics** | **Current smoker** | | ***p*** |
| --- | --- | --- | --- |
|  | **Yes** | **No** |  |
| **Vascular function** |  |  |  |
| VRI | 0.99 ± 0.61 | 1.02 ± 0.57 | 0.639 |
| Average ABI | 1.03 ± 0.15 | 1.06 ± 0.15 | 0.094 |
| **Biomarkers^a^** |  |  |  |
| ICAM-1 (ng/mL) | 244 (211–289) | 218 (168–266) | 0.004* |
| VCAM-1 (ng/mL) | 2,988 (2,729–3,487) | 3,072 (2,751–3,430) | 0.805 |
| ADMA (μmol/L) | 0.57 (0.50–0.72) | 0.58 (0.51–0.70) | 0.974 |
| ET-1 (pmol/L) | 0.40 (0.28–0.62) | 0.43 (0.27–0.57) | 0.830 |
| IL-6 (pg/ml) | 7.11 (4.14–10.98) | 5.73 (3.67–9.06) | 0.181 |

VRI, vascular reactivity index; ABI, ankle–brachial index; ICAM-1, intercellular adhesion molecule-1; VCAM-1, vascular cell adhesion molecule-1; ADMA, asymmetric dimethylarginine; ET-1, endothelin-1; IL-6, interleukin-6.

^a^ n = 262

**p* < 0.05 was considered significant.

**Table S2. Spearman’s correlation of ECW/TBW ratio with HTN, renal function, VRI, ABI, endothelial markers, and interleukin-6.**

| **Variable** | **ECW/TBW ratio** | |
| --- | --- | --- |
|  | ***r*** | ***p*** |
| HTN | 0.101 | 0.038* |
| eGFR (mL/min) | -0.161 | 0.001* |
| UPCR (g/g) | 0.129 | 0.008* |
| VRI | -0.114 | 0.020* |
| Average ABI | -0.194 | <0.001* |
| **Biomarkers^a^** |  |  |
| ICAM-1 (ng/mL) | 0.089 | 0.149 |
| VCAM-1 (ng/mL) | 0.111 | 0.073 |
| ADMA (μmol/L) | 0.133 | 0.031* |
| ET-1 (pmol/L) | 0.173 | 0.005* |
| IL-6 (pg/ml) | 0.133 | 0.031* |

ECW/TBW, extracellular water/total body water; HTN, hypertension; eGFR, estimated glomerular filtration rate; UPCR, urine protein/creatinine ratio; VRI, vascular reactivity index; ABI, ankle–brachial index; ICAM-1, intercellular adhesion molecule-1; VCAM-1, vascular cell adhesion molecule-1; ADMA, asymmetric dimethylarginine; ET-1, endothelin-1; IL-6, interleukin-6.

^a^ n = 262

**p* < 0.05 was considered significant.

**Table S3. Multivariate logistic regression analysis of factors associated with sarcopenia in patients with CKD (n = 420), further adjusting for ECW/TBW ratio.**

| **Variable** | **Multivariate** | |
| --- | --- | --- |
|  | **OR (95% CI)** | ***p* value** |
| Age (years) | 1.12 (1.08–1.16) | <0.001* |
| BMI (kg/m^2^) | 0.68 (0.61–0.76) | <0.001* |
| Average ABI (per 0.1 increase) | 0.81 (0.67–0.98) | 0.033* |
| VRI (per 0.1 increase) | 0.93 (0.88–0.98) | 0.010* |

In the multivariate models, age, gender, BMI, DM, albumin, eGFR, UPCR, pulse pressure, average ABI, VRI, and ECW/TBW were adopted.

CKD, chronic kidney disease; ECW/TBW, extracellular water/total body water; BMI, body mass index; ABI, ankle–brachial index; VRI, vascular reactivity index

**p* < 0.05 was considered significant.

**Table S4. Correlation matrix of vascular function tests, endothelial markers, and interleukin-6.**

| **Variables** | **Spearman’s correlation coefficients** | | | | | | |
| --- | --- | --- | --- | --- | --- | --- | --- |
|  | VRI | Average ABI | ICAM-1 (ng/mL)**^a^** | VCAM-1 (ng/mL)**^a^** | ADMA (μmol/L)**^a^** | ET-1 (pmol/L)**^a^** | IL-6 (pg/ml)**^a^** |
| VRI | – | – | – | – | – | – | – |
| Average ABI | 0.251* | – | – | – | – | – | – |
| ICAM-1 (ng/mL)**^a^** | -0.013 | -0.031 | – | – | – | – | – |
| VCAM-1 (ng/mL)**^a^** | -0.131* | -0.061 | 0.442* | – | – | – | – |
| ADMA (μmol/L)**^a^** | -0.155* | -0.044 | 0.039 | 0.312* | – | – | – |
| ET-1 (pmol/L)**^a^** | -0.183* | -0.108 | -0.008 | 0.152* | 0.161* | – | – |
| IL-6 (pg/ml)**^a^** | -0.111 | -0.071 | 0.063 | 0.176* | 0.243* | 0.209* | – |

VRI, vascular reactivity index; ABI, ankle–brachial index; ICAM-1, intercellular adhesion molecule-1; VCAM-1, vascular cell adhesion molecule-1; ADMA, asymmetric dimethylarginine; ET-1, endothelin-1; IL-6, interleukin-6.

^a^ n = 262

**p* < 0.05 was considered significant.
